# Fipronil 1% pour-on: further studies of its effects against lab-reared *Glossina palpalis gambiensis*

**DOI:** 10.1007/s00436-017-5599-3

**Published:** 2017-09-21

**Authors:** B. Sawadogo, J. B. Rayaisse, H. Adakal, A. T. Kabre, B. Bauer

**Affiliations:** 1 CIRDES, 01 BP 454, Bobo-Dioulasso, Burkina Faso; 2 Institut du Développement Rural, Université Polytechnique de Bobo-Dioulasso, Bobo-Dioulasso, Burkina Faso; 3 Université Dan Dicko Dan Koulodo de Maradi, BP 465, Maradi, Niger; 4 Institute for Parasitology and Tropical Veterinary Medicine, Freie Universitaet Berlin, Robert-von-Ostertagstr. 7-13, 14163 Berlin, Germany

**Keywords:** Fipronil, Tsetse flies, Survival, Persistence

## Abstract

In order to assess the residual effects of fipronil 1% on tsetse fly survival, male *Glossina palpalis gambiensis* were released on non-treated and treated cattle, with 0.1 ml of fipronil/kg b.w. as a pour-on formulation. In a second trial, the female fecundity performances were evaluated by feeding teneral females on the same cattle. These females were then mated and their production parameters monitored, as well as the survival of freshly emerged flies. Fipronil had a significant effect on tsetse fly survival (*p* < 0.001). Over a period of 30 days, up to 40% of tsetse fly mortality was observed within 72 h after tsetse were released. The residual effects ranged between 51 and 74 days when tsetse flies were released twice within a 15-day interval in the presence of a treated animal. When tsetse flies were fed on treated cattle through a parafilm membrane, 92 days after the treatment, no significant effect of fipronil was observed on the reproductive performance of females, i.e., as well as on fecundity (*p* = 0.948) and emergence rates (*p* = 0.743), or puparial weight (*p* = 0.422). This was also the case for the survival of young flies, with no difference observed between the two groups. After this study, it is confirmed that fipronil is highly effective against tsetse flies. Its efficacy in controlling ticks is already known but other externalities such as the control of biting insects add value to its use.

## Introduction

Vector control has been a major component for the control of human and animal African trypanosomosis. In addition to the usual deployment of impregnated traps/targets (Cuisance and Politzar 1983; Cuisance et al. 1994; Bauer et al. 1999; Courtin et al. 2015), topical application of insecticides has also been used to control tsetse and other biting flies (Bauer et al. 1992). Topical application is especially appreciated by farming communities, as it is directly applied on the animal (Kamuanga et al. 2001) and can kill as well tsetse flies as other hematophagous insects and ticks, with no need of costly installations.

However, the non-respect of insecticide prescription (doses and frequency of treatment) can lead to poor effects or loss of the animal. Therefore, there is a need to determine the treatment frequency in order to offer adapted recommendations to farmers. These requirements are already known for insecticides like deltamethrin (up to 90 days, Bauer et al. 1995). Our current study aimed to determine the efficacy and the residual effects of a topical application of fipronil 1% on tsetse fly survival and fecundity. Fipronil is acting at the level of the ligand-gated chloride channels in insects (Hunter et al. 1994). It is controlled by the neurotransmitter gamma-aminobutyric acid (GABA), interfering with the pre- and post-synaptic transfer of chloride ions across cell membranes. Since previous work by Bauer and Baumann (2015) showed long-lasting effects on survival of exposed *Glossina palpalis gambiensis*, we put particular emphasis on assessing eventual additional effects on the reproductive performance of tsetse flies.

## Material and method

### Topical application of fipronil on cattle

Six young male zebu cattle with an average weight of 100 kg b.w. were used. Seven (7) days prior to the beginning of the experiment, three cattle were treated on the back with fipronil at the dose of 0.1 ml/kg b.w., while the remaining three served as controls. The insecticide was expected to largely spread on the body surface. Two days after treatment, and in order to mimic natural conditions, treated and control animals were exposed during 3 h to sun and watered with 50 l of water. Exposure to sun and watering were repeated every 2 days as described by Bauer and Baumann (2015). The animals were maintained in two separate pens.

### Measuring tsetse fly survival

The experiment was performed in fly-proof facilities, subdivided in two pens with inner dimensions of 2 m height, 4 m in length, and 2 m in width. All animals were placed in the pens following a randomized rotation avoiding any bias.

First tsetse releases took place 7 days after their treatment. For each release, two cattle (one treated and one control) were introduced each in a pen, respectively, and then 102-day-old tsetse flies were released in each pen for 2 h. Each pen was humidified 1 h prior to the tsetse fly release, thus ensuring an adequate humidity in the different pens. After 2 h of exposure, all flying insects were caught with a hand net. They were then separated into engorged and non-engorged flies. Maintenance was ensured in an insectary at 25 °C and 75% relative humidity. Paralyzed flies were separated 2 h after their collection. Tsetse mortality was then daily assessed during 2 weeks, while they were fed through membranes with bovine blood collected from the abattoir of Bobo-Dioulasso, (Burkina Faso), as in the CIRDES main insectary. After 15 days of monitoring, surviving flies were again released in the presence of one treated or untreated animal. Releases took place until the mortality of the flies in the experimental groups remained below 50% during five consecutive releases.

### Assessing tsetse fly fecundity

This test was performed once mortalities of below 50% were recorded for five consecutive exposures. It was repeated three times (92 days after treatment, and then 15 and 30 days later). For each repetition, two groups of 100 females each (treatment and control) were used. Each group was subdivided into three Roubaud cages (13 cm × 8 cm × 5 cm). One day after emergence, the tsetse flies were exposed on the respective animal for feeding through a parafilm membrane thereby preventing any contact between the tsetse fly tarsi and the cattle body. Mated females were fed 6 days/week (3 days on treated/untreated animals, respectively) and for 3 days with the usual in vitro feeding system. During the 10 min feeding, a black piece of cloth covered the cage, so minimizing disturbance due to light. Tsetse were then maintained at 25 °C and 75% H° and mated the subsequent day with 6-day-old males, at a ratio of three females for one male. For each group, pupae were daily collected, counted, and weighed. After emergence, each tsetse fly was individually kept in a cage without any feeding and its survival recorded.

### Data analysis

Data on tsetse fly survival were analyzed under R 3.0.3 (R Development Core Team, 2013), while analyses on fecundity were run with Statistica, version 7.1. The Kaplan–Meir (Kaplan and Meier 1958) non-parametric estimator was used for the survival analysis, and the Cox model (Cox 1972; Cox and Oakes 1984), used to determine on one hand, the statistical significance of fipronil and time post treatment effect on tsetse fly survival and on the other hand, the interaction between the treatment and the post-treatment time. Comparison between dead, non-engorged, and gorged flies for each treatment was done using the *X*_2_ test of Pearson. The Student *t* test was used to compare the different variables collected during the test on the effect of the fipronil on tsetse fecundity.

## Results

### Effect of fipronil on tsetse fly survival

Up to 1800 tsetse flies were released during this experiment from which 1744 were recaptured (889 for the control and 855 for the treatment) and monitored daily during 30 days (15 days after the first release and another 15 days after the second release). Fipronil had a significant impact on tsetse flies’ survival (*p* < 0.0001) and the survival index of control flies I was two times higher than those fed on the treated animal (Table 1).

**Table 1:** Effect of fipronil on tsetse fly survival

Survival parameters	Control	Treated
Average survival (in days)	25.95	15.15
Index of survival	0.73	0.32
Median of survival or L50^a^	NA	51
Mortality relative risk	2.52	

*NA* not available

^a^L50: lethal 50: time at which up to 50% of flies were dead within the monitoring period

During the 30 days of monitoring, the average lifespan of control flies was 25.95 days, versus 15.15 for treated flies, hence a reduction of almost 50%.

**Fig. 1:**
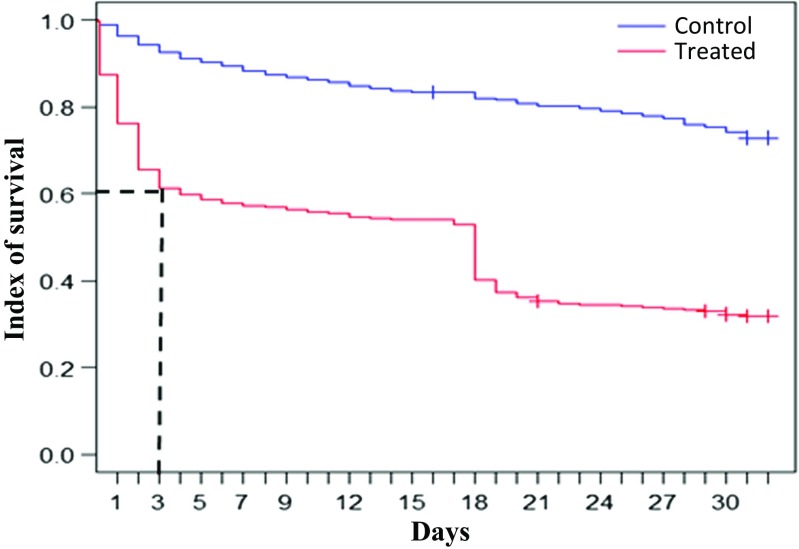
Overall survival graphic of treated and control tsetse flies during 30 days of monitoring

**Fig. 2:**
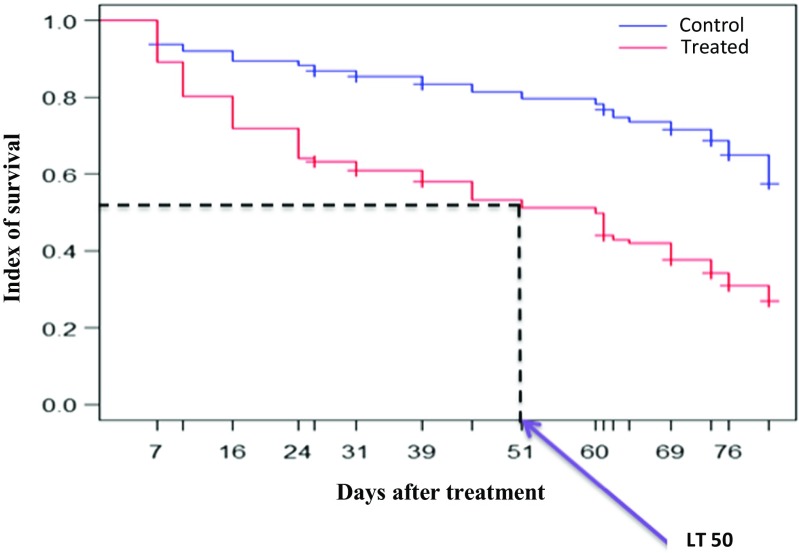
Tsetse fly overall survival within 81 days of monitoring. TL50: lethal 50: time at which up to 50% of flies were dead within the monitoring period

Mortality of tsetse flies fed on treated cattle was more relevant during the three first days, (particularly after the second day following blood uptake): up to 40% were dead (Fig. 1). The lethal 50 time (see Table 1), for tsetse flies fed twice on a treated animal was 51 days (Fig. 2).

**Fig. 3:**
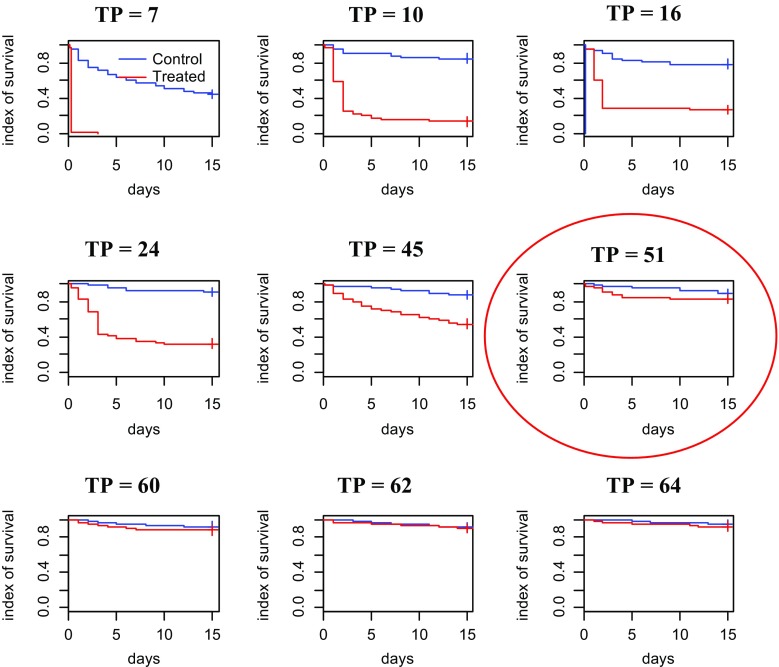
Tsetse fly initial survival (TP = time post treatment)

**Fig. 4:**
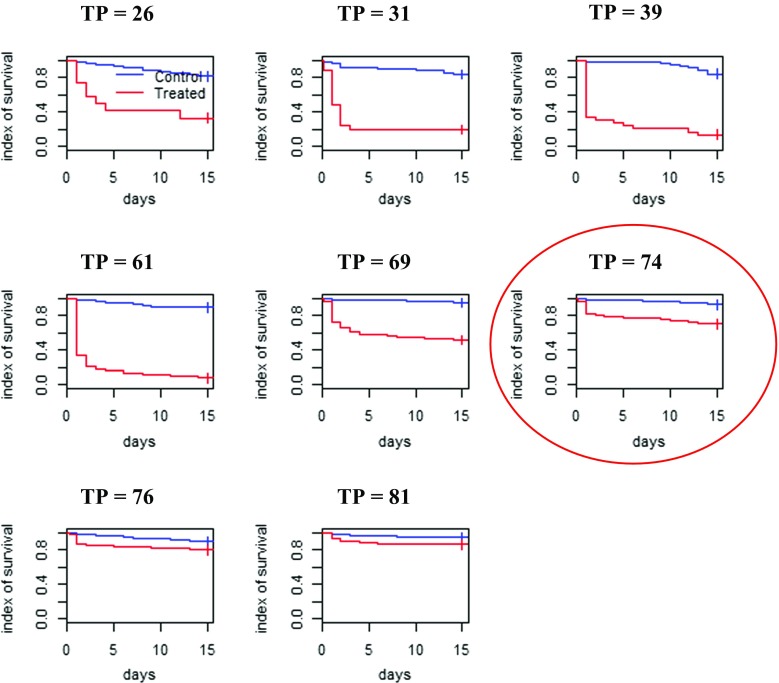
Tsetse fly survival—second release of survivors from initial release (TP = time post treatment)

There is a significant interaction between the variable “treatment” and the variable “days after treatment.” The days after treatment influence the average survival time of flies, while survival of control tsetse flies is independent from time. Between 0 to 41 days after treatment, the survival rates of the flies fed on control and treated cattle were significantly different (*p* < 0.0001). The residual effect of fipronil was monitored when tsetse flies were released for the first time on treated cattle, and then after the second release 15 days later for the remaining, surviving flies. For the first release, the survival indices between flies fed from treated and untreated animal were significantly different until the 51st days after treatment, after which no difference was observed (*p* = 0.193, Fig. 3). After the second release, a difference was again observed between the two groups until the 74th day after treatment, after which no difference was observed (Fig. 4).

### Comparison of fecundity for parental female flies

**Fig. 5:**
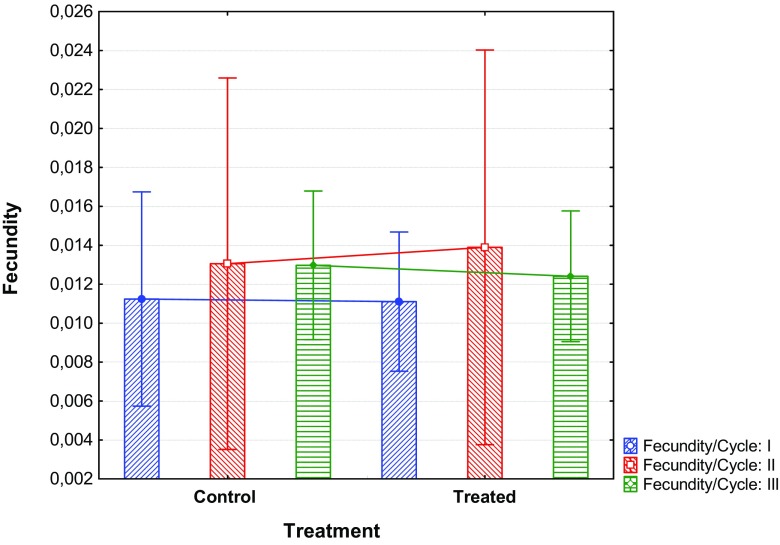
Fecundity of tsetse flies fed on untreated (control) and treated bulls, respectively, expressed as mean puparia/female/reproductive cycle during three consecutive reproductive cycles

For all the three repetitions (92, 107 and 121 days after treatment), no significant differences were observed between the flies fed on treated and control animals (Fig. 5).

Also, mean puparial weights as well as abortion and emergence rates were comparable in both groups.

## Discussion

### Effect of fipronil on tsetse fly survival

Exposures to fipronil-treated cattle significantly reduced tsetse fly survival, with a higher mortality risk of 2.52 (Table 1) for flies feeding on treated animals.

For up to 4 h after contact and blood uptake, no high mortalities were recorded, raising the presumption that fipronil had indeed a paralyzing effect after contact; however, most tsetse were killed after ingestion of the product.

The residual effect of fipronil lasted for a period of up to 74 days in our experimental setup and was exceeding most of the available pour-on formulations. Bauer and Baumann (2015) even recorded important mortalities 140, 170, and 190 days after treatment. However, contrasting with the present study, these results followed triple releases or triple feeding of caged tsetse flies on treated livestock. Contrary to the present study where flies had the opportunity to feed on the in vitro system between their exposures, all flies were only in contact with treated or untreated animals during the previous trial. Considering these results, it is likely that higher mortalities would have occurred if, in this study, tsetse flies had been given the opportunity to feed more often on treated livestock as well. Hence, the persistence would have considerably exceeded the observed 74 days. In the previous work, Bauer and Baumann (2015) concluded that fipronil did not prevent tsetse flies from feeding. Transmission of trypanosomes may therefore continue. Protection of valuable livestock by chemotherapy and/or use of a repulsive pyrethroid could overcome this initial phase, since it is assumed that, as a consequence of the campaign, a large proportion of tsetse flies will disappear.

### Effect of fipronil on the fecundity of female tsetse flies

As noted, fipronil had no effect on female tsetse fly reproductive performances, 90 days after treatment. Possible explanations are as follows:

- Fipronil does not act on tsetse fly fecundity, but it has to be noted that any impact on fecundity could have been masked by early mortalities
- The duration after treatment (92 days, following five successive survival rates above 50%) was too long. The amount of active ingredient could have been too low for any impact on fecundity. A more accurate assessment would have been possible if the tsetse flies were given the opportunity to feed three times during 1 week. This regimen would have been much closer to natural conditions.
- Eventually, differences could have been detected while conducting this trial simultaneously with the survival test.

Petit (2002) described fipronil as a non-systemic product that was working by contact. This is contrasting with our observations and also with work from other authors. Indeed, Davey et al. (1998), studying the effects and persistence of fipronil against *Boophilus microplus*, concluded that ticks needed to engorge on their host before impacts of the treatment could be expected. Bauer and Baumann (2015) also concluded that fipronil had a systemic effect. Additional work by Bauer (unpublished) showed high mortalities in tsetse flies when they were fed on blood drawn from a treated bull in an in vitro system. In the present study, significantly higher mortality rates were also found in engorged as compared to non-engorged tsetse flies. As a matter of fact, the withdrawal time for meat, as recommended by the manufacturer, is given as 100 days, which supposes that, effectively, there may exist a systemic effect. Fipronil is acting at the level of the GABA receptors; this mode of action is also germane to neonicotinoids, although thiametoxam has shown important transitory effects after fleeting contacts with a treated surface of *Musca domestica* (Bauer, unpublished). But the main effects of fipronil and neonicotinoids are occurring after ingestion of the products by insects.

We failed to detect, in our study, any effect of fipronil on tsetse fly fecundity, but Davey et al. (1998) found that it affected the fecundity of *B. microplus*, through a reduction of the reproduction index (99.7%), and no possibility of new larval infestation until up to 8 weeks after treatment. Petit (2002) found that fipronil increased puparial production but decreased the emergence rate. We, however, detected none of these effects in this study.

## Conclusions

From what was observed during the first part of the present study, it was evident that fipronil distinctly reduced tsetse fly survival (*Glossina palpalis gambiensis*), thereby confirming what had already been experienced for other arthropods like ticks. Based on what we have observed, fipronil could be used as follows for tsetse fly and tick control:

- The strategic use of fipronil for treatment of herds in tsetse fly infested areas is likely to reduce the target populations but may not reduce trypanosome infections during the initial phase of its application, since it has to be acknowledged that fipronil has at best a negligible repellent effect against tsetse flies; hence, transmission will occur at the first stage of an eventual campaign,
- Fipronil constitutes an additional tool for the control of trypanosomosis, and even other vector borne diseases. For tsetse fly control, an application at intervals of at least 6 months is recommended when considering the persistence observed by Bauer and Baumann (2015),
- As such, it is expected that its acceptance by herd owners will be high.

In view of its withdrawal time (100 days for meat), there is an obvious need to leave the application of the product exclusively in the hands of qualified veterinary staff. Care should also be taken that the product is not used on milking cows.
